# Using standardized patients to train telephone counselors for a clinical trial

**DOI:** 10.1186/1756-0500-7-341

**Published:** 2014-06-05

**Authors:** Erin S Rogers, Colleen Gillespie, Sondra Zabar, Scott E Sherman

**Affiliations:** 1 VA NY Harbor Healthcare System, 423 East 23rd Street, New York, NY 10010, USA; 2 Department of Population Health, New York University School of Medicine, 227 East 30th Street, New York, NY 10016, USA; 3 Division of General Internal Medicine, New York University School of Medicine, 550 1st Avenue, New York, NY 10016, USA

**Keywords:** Standardized patient, Clinical trial, Feasibility, Implementation, Behavior change

## Abstract

**Background:**

Standardized Patients (SPs) are actors trained to portray health care patients during the training and assessment of health care providers. This paper describes the methods and costs associated with using SPs to evaluate the skills of telephone counselors working on a clinical trial that evaluated a telephone smoking cessation program tailored for smokers using Department of Veterans Affairs mental health clinics.

**Findings:**

Conducting the SP exercises required five main steps: (1) Write a SP case description detailing patient demographics, demeanor, clinical symptoms and history, and instructions on how to respond to counseling, (2) Identify, select and train actors to portray the SP cases; (3) Conduct audio-taped counseling encounters between the SPs and counselors, (4) Rate the counselors on their core counseling competencies, (5) Provide feedback to counselors. The SPs and study supervisors reported that the checklist was easy to use when rating the counselors. Counselors reported that the SP encounters were realistic and helpful for practicing their clinical work and for building self-efficacy for working with real patients. The labor costs of developing two SP cases and training two SP actors was approximately $1,475. The per-session labor cost of conducting a 1-hour counseling session between one SP and one counselor was approximately $314.

**Conclusions:**

Using SPs to train telephone counselors working on a clinical trial was feasible and offered training benefits beyond those provided by didactic instruction and role plays. Our research group is now routinely using SPs for the training of incoming telephone counselors.

## Background

Standardized Patients (SPs) are actors trained to consistently portray health care patients based on well-developed clinical scenarios [1,2]. SPs are often used in the training and assessment of clinicians and have been used during the implementation of clinical trials to improve study procedures prior to working with real patients [3-5]. This paper describes the methods and costs associated with using SPs to evaluate the skills of telephone counselors working on a study that evaluated a telephone smoking cessation program for smokers using Department of Veterans Affairs (VA) mental health clinics. The goal of the parent study was to evaluate the effectiveness of an intensive telephone smoking cessation counseling program for VA mental health patients that included behavioral change counseling and problem solving therapy approaches [6-8].

## Methods

The parent study received approval from the Institutional Review Boards and Research and Development Committees at participating VA facilities. The study employed two counselors who underwent 15 hours of initial protocol training using didactic instruction and role-plays. After completing lectures and role-plays, the counselors completed two SP encounters. Developing and conducting the SP encounters required five main steps (Table 1).

**Table 1:** Steps required to conduct an SP exercise

**Step**	**Resources required**
1. Develop the SP case	• Case materials from a local school of medicine or professional accreditation bodies (e.g., AAMC)
	• Expert input for adapting or developing a case
2. Identify, select and train SP actors	• List/pool of potential actors
	• 2–3 hour training session
	• Actor payment (local rates)
3. Conduct the SP encounter	• Scheduled encounters between SPs and trainees
	• Audio or video recorder
	• Actor payment
	• Debriefing session (optional)
4. Rate trainee performance	• Standardized trainee assessment form and anchors
	• Actor payment (if SPs will rate trainees)
5. Provide feedback to trainee	• One-on-one or group feedback session with trainees
	• Feedback can come from SP and/or supervisor
	• Actor payment (if SPs will debrief with trainee)

SP = standardized patient.

AAMC = American Association of Medical Colleges.

### Step 1 - Develop the SP cases

Each counselor was to complete two SP encounters, so we developed two SP cases using our affiliation with New York University School of Medicine (NYUSoM). NYUSoM has a bank of SP cases, about a third of which focus on promoting behavior change. From NYUSoM’s case bank we selected two cases to adapt for our study. The first was a smoking cessation case, to which we incorporated a history of depression. The second was a post-traumatic stress disorder (PTSD) case, to which we incorporated a history of smoking. To further adapt the cases for a VA population, we made one case a Caucasian male in his 20s who served in Iraq and the other case an African American male in his 60s who served in Vietnam. Table 2 provides an overview of case #2. The study’s clinical supervisor, a VA clinical psychologist, wrote an extensive smoking, military, and mental health history for each case, and a careful description of how to respond to counseling questions, counselor tone and demeanor, and the counselor's proposed cessation treatment plans.

**Table 2:** SP case #1

**Case element**	**Example**
SP demographics	60 year-old African American male. Married for 28 years. Divorced for 7 years. Two children (ages 26 and 30). Unemployed for 8 years. Receives all health care from the VA.
SP demeanor	Friendly and open to cessation counseling but reserved when speaking about his military history.
SP clinical diagnosis, history, and symptoms as necessary	Smoking: Began smoking at age 19. Smokes 10–15 cigarettes per day. Used to smoke up to 2 packs per day. Decreased his smoking about 10 years ago when cigarettes began to cost more. Likes to smoke for relaxation. Has tried to quit a few times in the past. Most quits were “cold turkey.” Has tried the nicotine gum. Has never tried counseling to quit smoking. If asked on a scale of 0–10 his motivation to quit smoking, patient will report a 7. Motivated to quit smoking because it is too expensive and because he knows it is bad for his health. If asked on a scale of 0–10 his confidence in being able to quit, patient reports a 5. He is not very confident because of his struggles with stress and his previous relapses. He is unsure if he will be able to cope with his PTSD and stress without cigarettes.
	Mental health: PTSD linked to military service during Vietnam war. Suffers from insomnia, flashbacks of his best friend being killed in front of him, and heightened startle reflex to loud noises. History of alcohol abuse but sober for 3 years. Recovered from alcohol abuse using AA (which he still attends) and religion (Catholic). Very proud of his sobriety.
Other SP psychosocial history	Wife left him 7 years ago due to his drinking. Does not have great relationship with his adult children and would like to be closer to them. They live in NYC but he rarely sees them. Lost his last job (NYC bus driver) due to his drinking. He has many friends through AA and VA programs. Most of his friends smoke and may not support his quitting.
Instructions on how to respond to intervention competencies demonstrated by trainee during the SP encounter	Begins call in “contemplation” stage of change: thinking of quitting but not yet ready to set a quit date or commit to quitting. If the counselor uses appropriate counseling techniques (see protocol overview), the patient can commit to quitting. If the counselor does not use appropriate counseling techniques, the patient will remain unsure of his motivation to quitting.

### Step 2 - Identify, select and train standardized patient actors

Because our study population was Veterans with mental health disorders, playing a study participant would be emotionally demanding and challenging [9]. Therefore, we employed professional SP actors to accurately convey emotions and behavioral symptoms common in mental health patients. To locate SPs, we used NYUSoM’s pool of experienced SPs. Following recommendations in the literature for SP recruitment [2], we contacted actors who had experience playing the two cases adapted for the study and who matched the gender, age and racial demographics of the cases. After hiring two SPs, we emailed the assigned cases to each SP. The SPs reviewed their case prior to an in-person group training session with the study’s project director (ESR) and one of the study’s co-investigators (CG) who has SP training experience. The in-person training lasted 3 hours and provided the SPs with background information about the study and its counseling protocols (including a discussion of how to respond when the study counselors do and do not use appropriate counseling techniques), a detailed overview of each case, the opportunity to ask questions, and training in how to assess the study’s counselors using a checklist developed for the study (see Step 4 for checklist details).

### Step 3 - Conduct the SP encounters

The study’s project director scheduled telephone appointments for the counselors and SPs, who were located in separate rooms in the hospital. The counselors called the SPs and completed a first telephone counseling session using the methods they would use with study participants. We audio-taped the sessions using a digital recorder connected to each counselor’s phone, and the study co-investigator leading the SP encounters listed-in “live” on each call.

### Step 4 - Rate trainee performance

**Figure 1:**
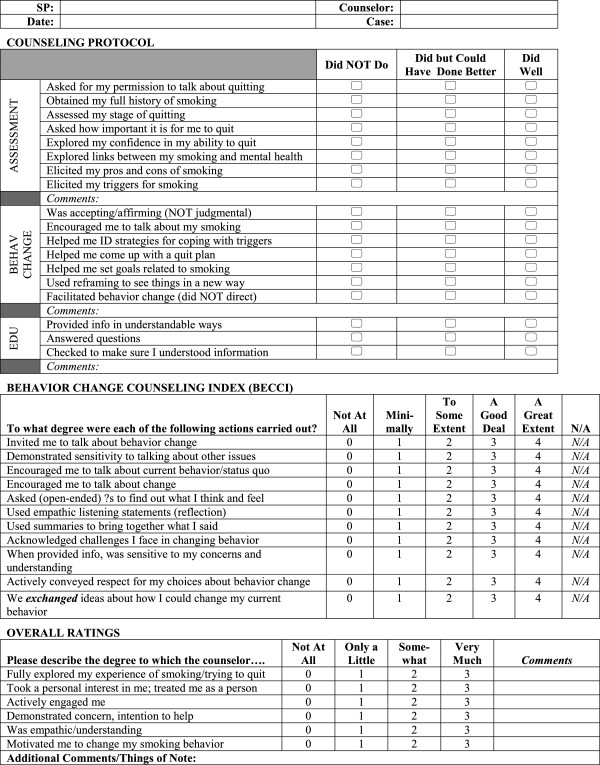
Counseling assessment form (SP Version).

SPs and trainers can consistently and accurately rate trainee performance with well-developed assessment tools [10,11]. Therefore, immediately after the counseling sessions, the SPs and the co-investigator present at the SP encounters rated the counselors using a 35-item checklist created for the study (Figure 1). To create the checklist we combined the validated Behavioral Change Counseling Index [12] with items specific to our study’s counseling protocol. Using Likert-type scales, the SPs and co-investigator scored the presence and quality of smoking cessation counseling behaviors outlined in the study’s counseling manual and counseling behaviors in accordance with behavioral change counseling techniques [7]. At a later date the study’s clinical supervisor listened to the session audiotapes and completed the checklist for each session.

### Step 5 - Provide feedback to trainee

Immediately after the SP encounters, the co-investigator present at the encounters, the SPs, and the counselors met for a 30-minute debriefing session. The co-investigator and SPs provided counselors with feedback on their performance. The counselors discussed how they felt during and after the encounters, including their opinion on whether the encounters felt real to them and how the cases could be improved.

At a later date the clinical supervisor provided one-on-one feedback to each counselor using the checklists completed by the supervisor and SPs. The clinical supervisor also reviewed the checklists to certify each counselor as ready to work with real patients. To be ready to work with real patients counselors needed the following score patterns: 1) On the Counseling Protocol scale counselors needed a rating of “Did Well” on the majority of items and no ratings of “Did Not Do;” 2) On the BECCI counselors needed a rating of “A Good Deal” or better on the majority of items and no ratings of “Minimally” or lower unless an item was not applicable; and 3) On the Overall Ratings scale counselors needed a rating of “Very Much” on the majority of items and no ratings of “Not at all.”

### Assessing costs

We calculated the labor costs associated with developing and conducting the SP exercises. We structured these costs into two categories: development costs (steps 1 and 2) and per-session costs (steps 3–5). To calculate the development costs we estimated the number of hours it took for personnel to develop two SP cases and identify and train two SP actors. We multiplied these hours by personnel hourly rates in 2009 dollars (the year in which we conducted the exercises) and added NYUSoM’s fringe benefit cost. To these study personnel costs we added the costs of the two SPs, which were calculated as the SPs’ hourly rate multiplied the number of hours each SP spent in training.

For per-session costs we calculated labor costs associated with a 1-hour counseling session between one counselor and one SP. We provide estimates for one hour of counseling, because this was the recommended length of time it would take to complete an intensive first counseling session with our difficult study population. As with the development costs, we multiplied the numbers of hours each study staff member worked on an SP session by his/her hourly rate and added NYUSoM’s fringe benefit cost. SP costs were calculated as the SP’s hourly rate multiplied the number of hours an SP spent in counseling and performing trainee rating and feedback.

## Findings

During the debriefing session the SPs and supervisors reported that the checklist was easy to use when rating the counselors’ competencies, and the counselors reported that they found the ratings to be helpful in understanding how they performed during the SP exercises. Both counselors reported that the SP exercises were helpful for practicing their clinical work and for practicing the logistics of working within a research setting. Both counselors also reported that working with the SPs allowed them to release some nervousness and build self-confidence in working with real patients. The counselors reported that they found the SP encounters to be realistic but reported two major weaknesses in the SPs’ abilities to portray VA mental health patients. One SP was not familiar with common military terminology of his combat era, and a second SP was not able to let the counselor know if he was taking any psychiatric medications. These lapses in knowledge were salient to the counselors and affected their ability to perceive the encounters as real.

Based on the success of the SP exercises with our first two counselors, we used SPs to train three subsequent study counselors after making some changes to our methods. We modified the cases to include more information about each SP’s military history and ongoing mental health treatment. In addition, to more closely resemble the procedures that would be used with real patients, the counselors called the SPs at their homes, we did not have a study team member listening-in on each call, and we did not have the counselors meet their SPs for an in-person debriefing session. Instead, the SPs provided feedback over the phone immediately after their counseling call.

Table 3 provides a summary of the labor costs associated with developing and executing the SP exercises using our modified session methods described in the previous paragraph. The cost of developing the SP cases and training SP actors was approximately $1,475. The per-session cost of conducting a 1-hour counseling session between one SP and one counselor was approximately $314.

**Table 3:** Estimated development and per-session costs of executing the SP training exercise

**Development costs**	**Tasks**	**Personnel/SP**	**Hours**	**Hourly rate**	**Hourly total**	**Fringe**	**Total cost**
Step 1. Develop 2 SP cases	Identify appropriate case from NYUSoM case bank. Adapt the case for current study.	Clinical Psychologist	5	$38	$190	35%	$257
		Co-investigator with SP expertise	5	$38	$190	35%	$257
				**Step 1 Total**			**$515**
Step 2. Identify, select and Train 2 SP actors	Identify appropriate actors from NYUSoM SP pools. Contact SPs and invite to participate. Develop and conduct 3-hour training meeting with 2 SPs. 2 SPs read and study case on their own. Arrange for SP payments.	Project Director	5	$29	$145	35%	$196
		Co-investigator with SP expertise	10	$38	$380	35%	$515
		SP1	5	$25	$125	n/a	$125
		SP2	5	$25	$125	n/a	$125
				**Step 2 total**			**$961**
				**Total development costs**			**$1,475**
**Per session costs**^**a**^	**Tasks**	**Personnel/SP**	**Hours**	**Hourly rate**	**Hourly total**	**Fringe**	**Total cost**
Step 3. Conduct SP encounter	Schedule counseling appointment. Counselor calls SP and provides 1 hour of counseling per protocol. Counselor completes call documentation.	Project Director	0.5	$29	$15	35%	$20
		Counselor	1.25	$25	$31	35%	$42
		SP	1	$25	$25	n/a	$25
				**Step 3 total**			**$87**
Step 4. Rate trainee performance	Supervisor listens to audiotape. Supervisor and SP complete rating form.	Clinical Psychologist	1.5	$38	$57	35%	$77
		SP	0.5	$25	$13	n/a	$13
				**Step 4 total**			**$90**
Step 5. Provide feedback to trainee	SP provides feedback immediately after counseling call. Supervisor provides feedback in one-on-one meeting with counselor.	Clinical Psychologist	1	$55	$55	35%	$74
		Counselor	1.5	$25	$38	35%	$51
		SP	0.5	$25	$13	n/a	$13
				**Step 5 total**			**$138**
				**Total per session cost**			**$314**

SP = Standardized Patient, NYUSoM = New York University School of Medicine.

^a^Per-session costs are for 1 hour of counseling between 1 SP and 1 counselor.

## Discussion

The literature supports the use of SPs to train and assess clinicians [1,5]. Our study supervisors found the use of SPs to be effective in assessing counselor readiness to work with real patients, and the counselors reported that they found the SP exercises to be realistic and helpful for their training. Labor costs associated with the use of SPs for counselor training were modest and comparable to costs found in prior research piloting the use of SPs to train substance abuse counselors [9].

Our methods have several limitations. First, we told the counselors that they would be treating SPs, which could have affected the way in which they provided treatment [13]. Second, we only tested the counselors on a single first call with each SP rather than have them complete a full course of counseling with an SP. Third, because we did not assess the counselors’ competencies with our checklist before the SP encounters, we are not able to determine if the counselors’ skills improved as a result of the SP exercise. Fourth, the BECCI portion of our checklist has been validated in prior research, but the two sections of the checklist created for this study were not validated. Lastly, generalization of our findings may be limited. Our study took place in a city with a relatively high cost of living. Therefore, labor costs may be significantly higher than if the study were conducted in a different city. In addition, the per-session costs necessarily depend on the length of a counseling session. Costs would be lower to train counselors for briefer interventions.

In summary, the purpose of this paper was to describe the application of the standardized patient training methodology to the assessment of counselors working on a clinical trial. The application of the methodology was feasible, inexpensive and offered training benefits beyond those provided by didactic instruction and role plays. Our research group is now routinely using SPs for the training of incoming telephone counselors.

## Abbreviations

NYUSoM: New York University School of Medicine; PTSD: Post-traumatic stress disorder; SP: Standardized patient; VA: Department of Veterans Affairs.

## Competing interests

The authors declare that they have no competing interests.

## Authors’ contributions

SES was the Principal Investigator on the study and contributed to the design of the SP exercises. ESR was the Project Director on the study, contributed to the design and conduct of the SP exercises, and conducted the cost analyses. CG conceptualized and directed the SP exercises. SZ refined the analysis of the SP exercise and refined the cost analysis methods. All authors interpreted results, and read and approved the final manuscript.
